# Popular Treatise on the Teeth

**Published:** 1845-03

**Authors:** 


*Popular Treatise on the Teeth.*—We are gratified to learn that Dr. Robert Arthur, surgeon dentist, of Philadelphia, is preparing a popular treatise on the diseases and treatment of the teeth, and that it will soon be published. Dr. A. is a good writer, and in every respect well qualified for the task he has undertaken. We hope his book will be extensively read. Correct information upon this subject, is much needed by community at large. It is one upon which most persons are wholly ignorant, and so superficial is the knowledge which nine out of ten have, of the qualifications necessary to the successful practice of the dental branch of medicine, that a mere newspaper advertisement, especially if it have appended to it the names of half a dozen individuals, is conclusive evidence of competency to exercise the various duties belonging thereto. If people would take a little pains to inform themselves upon the subject, they would not be so ready to trust the management of their teeth to every one who may chance to offer his services as a dentist.—*Balt. Ed.*

